# Editorial

**Published:** 1862-11

**Authors:** 


### Editorial.

### WM. HOPPS ON TRIAL FOR MURDER.

### A MEDICO-LEGAL CASE.

In a medico-legal aspect, the trial of Wm. Hopps, now (Dec. 25th,) progressing in the Circuit Court, is of much interest. The following, copied from the report in the *Chicago Daily Times*, states very fairly the grounds on which the defence is made, while the testimony of Dr. McFarland will be read with special interest. The opinions expressed by Dr. McFarland, in relation to the mind of the prisoner, were fully sustained by all the medical witnesses, who were called on to testify in the case:—

Yesterday was the sixth day of the trial of William Hopps in the Circuit Court of this county, for the murder of his wife. The fact of the commission of the homicide by the prisoner has not been denied by counsel for the defence, but, upon the other hand, openly admitted. The defence rest their whole case upon the plea of insanity. The testimony has been tedious, by reason of its great length, and we trust is about drawing to a close. The defence have unquestionably proved the actions of the prisoner's mind at least very morbid and erratic. He seems to have labored under a delusion of his wife's infidelity, and has assigned for his belief the most frivolous reasons, having no bearing upon the case, but which, however, seemed to him the most conclusive proofs, establishing the truth of his suspicion beyond the possibility of a doubt. For a long series of years, commencing with a trivial lawsuit about a horse, he has imagined that his neighbors had combined against him, and that the Masons, of which order he was a member, were seeking his life. He did not know his most intimate friends upon some occasions, and was subject at times of mental depression or hilarity. His suspicion of his wife found vent in torrents of abuse, principally poured out during the night time. He was restless, uneasy, singular. From these things, and many others, including his manner, appearance, voice, incoherent conversation, rarely confining himself to one subject, but wandering among ideas suggested by the subject before his mind, his physical condition, pulse from ninety to one hundred and twenty, whereas it should be about seventy, his apparent want of realization of guilt, the insanity of his brother, and his own seeming justification of his crime, and other things occurring to medical men, upon a careful examination, have induced some of the most scientific men in the State, including the Superintendent of the State Insane Asylum, to pronounce him, in the most unequivocal manner, insane and incapable of exercising any judgment in the commission of the crime.

THE TESTIMONY OF DR. McFARLAND.

Andrew McFarland called and sworn upon the part of the defence:

"I have been a physician for the past twenty-five years, and am now the Superintendent and Physican of the Illinois Insane Asylum. The treatment and management of the insane is now the exclusive study of my life. I had two interviews last Wednesday with the prisoner; I was not introduced to him in my real capacity but as a mere casual caller. I made no special examination of the physical condition of the prisoner, but was struck with his remarkable outward appearance. There were but three expressions to his countenance, which were non-responsive to the spirit of his narrations. One expression of his face was blank solidity; another was expression of profound conclusion, mingled with inquiry, when he thought he had established a fact; the third expression was when he smiled. The prisoner has but one laugh, it cannot be called idiotic or either silly, but a fatuous laugh. The pupil of his eye is immoveable, neither expanding or contracting with the admission or exclusion of light. I believe it has passed beyond the control of his intellect, and only responded to the play of his passions, and I thought I could detect something fiendish in it. When put upon a train of ideas his mind seemed to labor with difficulty, and it travelled off to collateral facts as a matter of relief or rest. When I was introduced to him I expressed surprise that he could be a prisoner, and this made it incumbent upon him to tell me the circumstances of his arrest. He told me the various stories others have given to the jury—the commencement of his troubles at the time of the horse difficulty, the toast, the onions, the veal, and the circumstances at the Sherman House about the Masons, and they all struck me as the creations of his fancy. His voice, like his countenance, never changes, except when he has reached and expressed a conclusion of his wife's infidelity, when it dropped into a kind of pitiful monotone. After giving the names of several persons with whom he knew his wife had been unfaithful, he used the expression 'and, indeed, with almost everybody,' as if he thought her criminality was indiscriminate. The measure of the disease of the prisoner's brain may be classed under the single idea of an insane delusion, which is a belief in the reality of things which can have no existence, according to any probable experience or testimony; something in violation of universal experience. All definitions of insanity are more or less fallacious and inaccurate, on account of the peculiar disease to which they are applied, and in my definition of an insane delusion I do not mean that the facts which are believed to be true must in all cases be facts which cannot exist under any circumstances that are impossible; but that their existence, if depending upon the proofs the lunatic adduces for his belief, would be an impossibility or improbability. A delusion has an infinitely stronger effect upon the mind of the subject than the sober connection of a fact can have upon the mind of a rational person. Mr. Frenchazel, lately confined in the Illinois Insane Asylum, is a case in point. His mind was unimpaired, save in a single respect: he imagined the sheriff was after him for the purpose of hanging him. He was confined in the fourth story of the building, and the window of his room was secured by grating deemed to be sufficiently strong to resist the strength of any man. Frenchazel, laboring under his delusion, fancied the sheriff was ascending the stairs, and, tearing the grating from his window, threw himself out upon the brick pavement, and was, of course, killed by the fall. The conviction of the fact that the sheriff was approaching with just such intentions would not have impelled a sane man to throw himself from that window. I believe if a man was laboring under the delusion that it was necessary for him to burn up his house, he would lay it upon the fire without a second's hesitation. These delusions are as variable as the imagination of man, and there exists no possible combination of facts out of which they cannot be fashioned. Rarely any two delusions are alike. The prisoner may have two brothers like himself laboring under insane delusions; one may imagine himself an apostle sent to convert the world, while the other may imagine that he owns the Bank of England, but they all denote the same degree of insanity. The first will do nothing more than preach upon the street corners, the second may do nothing worse than squander his means, imagining that he has an inexhaustible mine upon which to draw after all has been spent. While the prisoner, whose delusion is that his wife is a prostitute, will commit homicide, and the same conclusion which would make the one brother a street preacher and the other a spendthrift, with their peculiar delusions, would make the prisoner a homicide with his. It would inevitably come sooner or later. The condition of the prisoner is somewhat remarkable: his delusion is clear and unmixed and runs completely through the texture of his mind, and yet his mind, on any matter disconnected with the subject of the delusion, is perfectly clear. The number of patients in the insane asylum with minds as clear as that of the prisoner is very few, but over three-fourths of the patients now under my charge have delusions less plainly marked and deep and less apparent in their manifestations than the delusion under which the prisoner is laboring. Mr. Frenchazel, to whom I have alluded, was afflicted with an insanity much like that of the prisoner; he was a man of more original intelligence and education; he bore an unblemished character; was accurate in his transactions of business; had acquired considerable wealth and had an accurate idea of the value of money and its relations to any article which he wished to sell or buy. He went insane from no known cause occurring at the time, but really from an hereditary disposition. An insane delusion that a wife was untrue, operating for years upon a nervous temperament, would sooner or later result in homicide. It might occur sooner or later, but it would eventually occur. The testimony I have heard in this case has confirmed the opinion I have expressed with reference to the prisoner. Homicides committed by insane persons are always marked by unusual atrocity and malignity. The action of the defendant is that of a man who feels justified in his own mind by the delusion which has taken possession of his faculties. A man who always has one fixed delusion when he becomes intoxicated, though it may never attend him when sober, may be regarded as insane and liable to deport himself as such at any moment. From my knowledge of insane persons

I could not be imposed upon in this case by stimulation or deception; this is a form of insanity which cannot be stimulated, unless the person attempting it is profoundly acquainted both with the subject of insanity and the arts of stimulation."

Here the court adjourned until 2 o'clock P. M., and at that hour reassembled and continued the examination of Dr. McFarland:

*Cross-Examination.*—"Delusions may be excited by stimulants, by ardent spirits, riding in the sun, or any other excitants, and there may be cases where the delusion is attributable to liquor. I am of the opinion that the prisoner's insanity is paroxysmal in its character, and is attended with an increased appetite for liquor. It is a well-known fact in asylums that in such paroxysms the subject has an increased desire for stimulants, and, unless they are given, he becomes worse. Dr. Allen gave me the first intimation I had of this case, gave me an outline of it, and asked what the probable expense would be of securing my attendance upon the trial. I made a calculation upon what my actual disbursements would be and replied accordingly. Fifty dollars is the sum I named; I deemed my time paid for by the people, and that I have no right to charge for that. Mr. McComas suggested to me that I had better not visit the prisoner alone. There is a difference between insanity and jealousy, but Othello's jealousy was carried beyond the bounds of reason. If the prisoner knew his act was wrong, though he may have committed it under an irresistable impulse, it is possible for him to have an idea of right and wrong in the abstract, and still in this particular act has been incapable of determining between right and wrong; in such case he should not be punished. If the prisoner had expressed any sincere regrets at the commission of the deed, I should regard it as an evidence that he knew it was wrong; taking measures to secrete the knife would be no indication of it. I do not think liquor alone induced the action or contributed to it; he would have done it quite as quickly sober or drunk. Liquor, by operating as an excitant for a term of years upon his constitution, may have brought him sooner into insanity, and in this way it may have contributed indirectly to bringing about the delusion under which he killed his wife, but, in causing the homicide at that particular time and place, it had but a secondary place, if one at all. He would have killed his wife any how; a homicide act is the natural result of such delusions. Had he been simply intoxicated at the time he would have expressed some contrition afterwards, and pride would not prevent him."

*By the Court.*—"Intellectual insanity is when the intellectual organs are affected, and consists in a disturbed state of thought with no particular moral misconceptions. The idea of moral insanity, in which the intellectual powers are not disturbed, is erroneous. There is usually more or less moral perversion with all cases of intellectual insanity. Liquor excites the passions, makes a person pugnacious or angry, but in the selection of objects upon which to vent itself it is indiscriminate and diffuse, as liable to select one as another, delusions is special, and any dislike springing from it attaches always to particular persons. There is no such fixedness in a drunken man. Liquor simply excites. A man laboring under a delusion may be impelled by an impulse which overthrows his will to do a deed he knows to be wrong."

*Direct examination resumed.*—"I heard some regrets from the prisoner myself, but they appeared to be of a general nature, such as regret that his wife had been untrue, regret that his life had been unhappy, regret at the grief into which his act had plunged his family, and regret that his wife's course had rendered necessary the commission of the deed. The act, as a righteous one, seemed ever to be justified in his mind. The condition of the pulse alone is of little importance, but when taken in connection with other facts becomes strong corroborative evidence."

P.S.—Since the above was in type, the trial has been brought to a close: and the jury returned a verdict of Guilty of Wilful Murder. The chief ground on which the jurors were induced to set aside the unanimous testimony of medical witnesses, was doubtless the belief that whatever mental impairment or derangement existed, had been the result of the long continued and inordinate use of intoxicating liquors A motion has been made for a new trial.

---

### AMERICAN MEDICAL ASSOCIATION.

---

"Since the commencement of the rebellion, with the exception of the American Medical Association, the medical societies, state and local, have exhibited commendable activity. They have all had their stated meetings, and the discussions have, in general, been unusually interesting. In our opinion, the National Association, the parent society, should have held its annual meeting, but the committee determined otherwise. The same reasons which then influenced them to adjourn the meeting another year, still exist, and in three-fold intensity."—*American Med. Times.*

The "reasons" that induced the Committee of Arrangements to notify an adjournment of the annual meeting, were simply the earnest solicitations of officers and members of the association, in almost every section of the country. Of a score of letters received by the committee from officers and prominent members of the association located in Boston, New Haven, New York, Troy, Albany, Philadelphia, Cincinnati, and St. Louis, only two were in favor of holding the annual meeting last June. The Committee of Arrangements are ready to provide for the meeting whenever the profession will attend. Shall we have the meeting next June? Will our professional brethern give us their views on the subject without delay?

---

Transactions of the New York Academy of Medicine.—We have received the advance sheets of Volume I. of this Society, containing several valuable papers; and an abstract of the discussions elicited by them. We shall notice them more fully hereafter.

---

Sick and wounded in Washington and vicinity.—There are at this date, 13,263 sick and wounded soldiers in the various hospitals in Washington, Georgetown, and Alexandria. In the hospitals at Frederick there are 2,985, and at Hagerstown and Harper's Ferry about 3,000.—*Philadelphia Medical & Surgical Reporter.*

---

Surgeon J. V. Z. Blaney, United States Volunteers, has been ordered to report to Gen. Viele, at Norfolk, Va.

---

Ass't-Surgeon C. C. Dumreicher, U.S.A., has arrived at San Francisco, Cal., *en route* to Camp Pickett, San Juan Island.

---

Provision for taking Care of the Wounded in Battle.—During the present rebellion, much complaint has been made on account of inadequate provisions for promptly taking care of the wounded during and after a battle.

The following order, issued under the direction of Gen. McClellan, before he was removed from the command of the army of the Potomac, will show that no such complaints need be made in future, if the order is obeyed with any degree of efficiency.

We also copy from the *Medical and Surgical Reporter*, the Surgeon-General's new bill of fare for hospitals:—

### FIELD HOSPITALS IN THE ARMY OF THE POTOMAC.

CIRCULAR.

HEADQUARTERS ARMY OF THE POTOMAC,
*Medical Director's Office, October* 30, 1862.

In order that the wounded may receive the most prompt and efficient attention during and after an engagement, and that the necessary operations may be performed by the most skilful and responsible surgeons at the earliest moment, the following instructions are issued for the guidance of the Medical Staff of this Army; and Medical Directors of Corps will see that they are promptly carried into effect:

Previous to an engagement there will be established in each Corps a Hospital for each Division, the position of which will be selected by the Medical Director of the Corps.

The organization of this Hospital will be as follows:

1st. A Surgeon in charge;
One Assistant-Surgeon to provide food, shelter, etc.;
One Assistant-Surgeon to keep the records, etc.

2d. Three Medical Officers to perform operations; three Medical Officers as Assistants to each of these Officers.

3d. Additional Medical Officers and Hospital Stewards and Nurses of the Division.

The Surgeon in charge will have general superintendence, and be responsible to the Surgeon-in-Chief of the Division for the proper administration of the Hospital.

The Surgeon-in-Chief of Division will detail one Assistant-Surgeon, who will report to, and be under the immediate orders of, the Surgeon in charge, and whose duties shall be to pitch the Hospital Tents and provide straw, fuel, water, blankets, etc.; and, when houses are used, to put them in proper order for the reception of wounded. This Assistant-Surgeon will, when this shall have been accomplished, at once organize a kitchen, using for this purpose the Hospital mess-chest and the kettles, tins, etc., in the ambulances. The supplies of beef stock and bread in the ambulances, and of arrow root, tea, etc., in the hospital wagon, will enable him to prepare quickly a sufficient quantity of palatable and nourishing food. All the Cooks, and such of the Hospital Stewards and Nurses as may be necessary, will be placed under his orders for these purposes.

He will detail another Assistant-Surgeon, whose duty it shall be to keep a complete record of every case brought to the Hospital, giving the name, rank, company, and regiment, the seat and character of injury, the treatment, the operation if any be performed, and the result; which will be transmitted to the Medical Director of the Corps, and by him sent to this office.

This officer will also see to the proper interment of those who die, and that the grave be marked with a *head-board*, with the name, rank, company, and regiment, legibly inscribed thereon. He will make out two "tabular statements of wounded," which the Surgeon-in-Chief of Division will transmit within thirty-six hours after a battle; one to this office, (by a special messenger, if necessary), and the other to the Medical Director of the Corps to which the hospital belongs.

There will be selected from the Division by the Surgeon-in-Chief, under the direction of the Medical Director of the Corps, three medical officers, who will be the operating staff of the hospital, with whom will rest the immediate responsibility of the performance of all important operations. In all doubtful cases they will consult together, and a majority shall decide upon the expediency and character of the operation. These officers will be selected from the Division without regard to rank, but *solely* on account of their known prudence, judgement, and skill. The Surgeon-in-Chief of the Division is enjoined to be specially careful in the selection of these officers, choosing only those who have distinguished themselves for surgical skill, sound judgment, and conscientious regard for the highest interest of the wounded.

There will be detailed three medical officers to act as assistants to each one of these officers, who will report to him, and act entirely under his direction.

It is suggested that one of these assistants be selected to administer the anæsthetic. Each operating surgeon will be provided with an excellent table from the hospital wagon, and with the present organization for field hospitals, it is hoped that the confusion and the delay in performing the necessary operations so often existing after a battle, will be avoided, and all operations hereafter be *primary*.

The remaining medical officers of the division, except one to each regiment, will be ordered to the Hospital to act generally as assistants and dressers.

Those who follow regiments to the field will establish themselves, each one at a temporary depot, at such a distance or situation in the rear of his regiment, as will insure safety to the wounded, where they will give such aid as is immediately required; and they are here reminded, that whilst no personal consideration should interfere with their duty to the wounded, the grave responsibilities resting upon them render any unnecessary exposure improper.

The Surgeon-in-Chief of the Division will exercise general supervision, under the Medical Director of the Corps, over the medical affairs in his Division. He will see that the officers are faithful in the performance of their duties in the Hospital and upon the field; and that, by the Ambulance Corps, which has heretofore been so efficient, the wounded are removed from the field carefully and with despatch. Whenever his duties permit, he will give his professional services at the Hospital, and will order to the Hospital, as soon as located, all the Hospital wagons of the brigades, the Hospital tents and furniture, and all the Hospital stewards and nurses. He will notify the captain commanding the Ambulance Corps, or, if this be impracticable, the first lieutenant commanding the division ambulances, of the location of the Hospital.

No medical officer will leave the position to which he shall have been assigned without permission; and any officer so doing will be reported to the Medical Director of the Corps, who will report the facts to this office.

Medical Directors of Corps will apply to their Commanders on the eve of a battle for the necessary guard and men for fatigue duty. This guard will be particularly careful that no stragglers be allowed about the Hospital, using the food, etc., prepared for the wounded.

No wounded will be sent away from any of these Hospitals without authority from this office.

Previous to an engagement a detail will be made by Medical Directors of Corps of the proper number of medical officers, who will, should a retreat be found necessary, remain and take care of the wounded. This detail the Medical Directors will request Corps Commanders to announce in orders.

The skilful attention shown by medical officers of this army to the wounded upon the battle fields of South Mountain, Crampton's Gap, and the Antietam under trying circumstances, gives the assurance that, with this organization, the Medical Staff of the Army of the Potomac can with confidence be relied upon under all emergencies to take charge of the wounded entrusted to its care.

*Blank form of Report to the Medical Director.*

TABULAR STATEMENT of wounded in at the day of , 186

| REGION OF BODY WOUNDED. | MISSILE. | | | | AMPUTATIONS. | | | | CHLOROFORM. | | TOTAL. |
|---|---|---|---|---|---|---|---|---|---|---|---|
| | Cannon Ball. | Shell. | Bullet. | Other. | Primary. | Secondary. | Exsections. | Other operations. | Given in. | Deaths from. | |
| Head, ................ | | | | | | | | | | | |
| Neck, ................ | | | | | | | | | | | |
| Chest, ................ | | | | | | | | | | | |
| Abdomen,................ | | | | | | | | | | | |
| Back and spine, ........ | | | | | | | | | | | |
| Hips and genitals, ..... | | | | | | | | | | | |
| Shoulder, ................ | | | | | | | | | | | |
| Arm, ................ | | | | | | | | | | | |
| Forearm and hand, .... | | | | | | | | | | | |
| Thigh, ................ | | | | | | | | | | | |
| Leg, ................ | | | | | | | | | | | |
| Foot, ................ | | | | | | | | | | | |
| OTHER OPERATIONS. | | | | | | | | | | | |
| TOTAL, ................ | | | | | | | | | | | ...... |

This report will be sent to the Medical Director of the Army (by special messenger, when practicable) within thirty-six hours after a battle.

................................................, *Surgeon,*

JONA. LETTERMAN,
*Surg. and Med. Director.*

---

*The Surgeon-General's New Bill of Fare for the Hospitals.*—The following diet table has been prepared by Surgeon-General Hammond, and the following order has been issued:—

"Medical officers who receive this Diet Table are directed to adopt it immediately in the hospitals under their charge, and to comply strictly and carefully with its provisions for thirty days, keeping during that period an accurate account of expenditure from the hospital fund. At the end of that time they will report the results of this experimental trial, its effects upon the sick and upon the hospital fund, and will make such suggestions as they may deem appropriate, the object being to test the practical operation of the Diet Table, before adopting it as a standard for the General Hospitals. It is recommended that the diets be prepared according to receipts in the Steward's Manuel.

*One Day Diet, Avoirdupois weight.—Full Diet.*

| | | |
|---|---|---|
| Meat, oz................16 | Indian Meal, oz....1·60 | Milk, oz..................8 |
| Bread, oz................18 | Salt, gill..................0·16 | Butter, oz.................1 |
| Potatoes, oz............ 8 | Coffee, oz.................0·80 | Flour, oz..................0·25 |
| Other vegetables, oz. 8 | Tea, oz.....................0·12 | Molasses, gill...........0·32 |
| Rice, Hominy, or | Sugar, oz..................2·40 | Vinegar, gill.............0·32 |

*Tuesday—in lieu of Fresh Meat.*

| | |
|---|---|
| Pork, oz................. 8 | Beans, gill..............0·64 |

*Half Diet.*

| | | |
|---|---|---|
| Meat, oz................ 8 | Indian Meal, oz...1·60 | Milk, oz..................8 |
| Bread, oz................16 | Salt, gill..................0·16 | Butter, oz.................1 |
| Potatoes, oz............ 6 | Coffee, oz.................0·80 | Flour, oz..................0·25 |
| Other vegetables, oz. 6 | Tea, oz.....................0·12 | Molasses, gill...........0·32 |
| Rice, Hominy, or | Sugar, oz..................2·40 | Vinegar, gill.............0·32 |

*Chicken Diet.*

| | | |
|---|---|---|
| Fowl, oz................12 | Tea, oz.....................0·24 | Milk, oz.................. 8 |
| Bread, oz................18 | Sugar, oz..................2·40 | Butter, oz................ 1 |
| Salt, gill.................0·16 | | |

*Milk Diet.*

| | | |
|---|---|---|
| Bread, oz................14 | Milk pt.................... 3 | Sugar, oz................ 1 |
| Rice, oz.................. 2 | | |

*Beef-Tea Diet.*

| | | |
|---|---|---|
| Beef (without bone) oz........................ 8 | Salt, gill.................0·32 | Sugar, oz................ 2 |
| Bread, oz................12 | Tea, oz....................0·24 | Milk, oz.................. 4 |

*Articles furnished on extra orders for special cases.*

| | | |
|---|---|---|
| Fish. | Oranges. | Corn Starch. |
| Oysters, raw. | Lemons. | Tapioca. |
| Oysters, stewed. | Fruits. | Crackers. |
| Clam soup. | Ice. | Toast, |
| Vegetables, (special). | Barley-water. | Chocolate. |
| Milk. | Rice-water. | Cocoa. |
| Sugar, white. | Jelly-water. | Blanc Mange. |
| Sugar, brown. | Lemonade. | Wine Whey. |
| Barley. | Veal Cutlet. | Brandy. |
| Cracked Wheat. | Ham, boiled. | Whiskey. |
| Beef Steak. | Poultry. | Wine, Sherry. |
| Beef Essence. | Game. | " ——— |
| Beef Extract. | Eggs. | Porter. |
| Mutton Chop. | Gruel, Corn Meal. | Ale. |
| Mutton Broth. | Gruel, Oat Meal. | Cider. |
| Wine Jelly. | Farina. | Milk Punch. |
| Custard. | | |

### *Low Diet.*

| | | |
|---|---|---|
| Meat, oz............... 8 | Sugar, oz............. 2.4 | Rice, Farina, Corn Starch, or Bread made into Pudding, oz............. 2 |
| Bread, oz............... 14 | Milk, oz................. 8 | |
| Salt, gill............... 0·16 | Butter, oz............ 1 | |
| Tea, oz.................0.240 | | |

### *Daily Meals—Full Diet—Sunday.—Breakfast.*

| | | |
|---|---|---|
| Coffee, pt................ 1 | Butter, oz............... ½ | Molasses, gill..........0·32 |
| Bread, oz................ 6 | Hominy, boiled, oz... 2 | |

### *Dinner.*

| | | |
|---|---|---|
| Roast Beef, oz.........16 | Other vegetables, oz. 8 | Rice Pudding.......... |
| Potatoes, oz............ 8 | Bread, oz................ 6 | |

### *Supper.*

| | | |
|---|---|---|
| Tea, pt.................. 1 | Bread or crackers, oz...................... 6 | Cheese, oz.............. 3 |

### *Monday.—Breakfast.*

| | | |
|---|---|---|
| Coffee, pt................ 1 | Butter, oz............... ½ | Cold Meat, oz.......... 4 |
| Bread, oz................ 6 | | |

### *Dinner.*

| | | |
|---|---|---|
| Beef Soup, pt.......... 1½ | Bread, oz................ 6 | Other vegetables, oz. 8 |
| Beef Soup Meat, oz..12 | Potatoes, oz............ 8 | |

### *Tea.*

| | | |
|---|---|---|
| Tea, pt.................. 1 | Bread, oz............... 6 | Butter, oz.............. ½ |

### *Tuesday.—Breakfast.*

| | | |
|---|---|---|
| Coffee, pt............... 1 | Butter, oz.............. ½ | Meat Hash with Vegetables, oz...... 8 |
| Bread, oz............... 6 | | |

### *Dinner.*

| | | |
|---|---|---|
| Pork / Beans } Baked, oz... 8 or in Soup, gill.....................0·64 | Potatoes, oz............ 8 | Bread, oz............... 6 |
| | Other vegetables, oz. 8 | Indian Pudding. |

### *Tea.*

| | | |
|---|---|---|
| Tea, pt.................. 1 | Butter, oz.............. ½ | Fruit, stewed, oz...... 4 |
| Bread, oz................ 6 | | |

### *Wednesday.—Breakfast.*

| | | |
|---|---|---|
| Coffee, pt............... 1 | Butter, oz.............. ½ | oz...................... 2 |
| Bread, oz................ 6 | Indian Meal, boiled | Molasses, gill..........0·32 |

### *Dinner.*

| | | |
|---|---|---|
| Beef recently corned, or Ham boiled, oz..16 | Potatoes, oz............ 8 | Bread, oz............... 6 |
| | Other vegetables, oz. 8 | Pickles, oz............. 1 |

### *Tea.*

| | | |
|---|---|---|
| Tea, pt.................. 1 | Bread, oz............... 6 | Butter, oz.............. ½ |

The above will serve as a sample of the full diet table, and the following of the

### *Half Diet.—Sunday.—Breakfast.*

| | | |
|---|---|---|
| Coffee, pt............... 1 | Butter, oz.............. ½ | oz...................... 2 |

| | | |
|---|---|---|
| Bread, oz............... 6 | Indian Meal, boiled, | Molasses, gill..........0·32 |

*Dinner.*

| | | |
|---|---|---|
| Beef Broth, pt......... 1 | Bread, oz............... 4 | Other vegetables, oz. 6 |
| Beef Broth Meat, oz. 8 | Potatoes, oz........... 6 | Rice Pudding. |

*Tea.*

| | | |
|---|---|---|
| Tea, pt................. 1 | Bread, oz............... 6 | Butter, oz.............. ½ |

*Monday.—Breakfast.*

| | | |
|---|---|---|
| Coffee, pt............... 1 | Bread, oz............... 6 | Butter, oz.............. ½ |

*Dinner.*

| | | |
|---|---|---|
| Beef Soup, pt.......... 1 | Bread, oz............... 4 | Other vegetables, oz. 6 |
| Beef Soup Meat, oz... 8 | Potatoes, oz........... 6 | |

*Tea.*

| | | |
|---|---|---|
| Tea, pt................. 1 | Bread, oz............... 6 | Butter, oz.............. ½ |

*Tuesday.—Breakfast.*

| | | |
|---|---|---|
| Coffee, pt............... 1 | Butter, oz.............. ½ | Molasses, gill..........0·32 |
| Bread, oz............... 6 | Hominy, boiled, oz... 2 | |

*Dinner.*

| | | |
|---|---|---|
| Mutton or Beef Broth pt...................... 1 | Bread, oz............... 4 | Other vegetables, oz. 6 |
| Mutton or Meat, oz.. 8 | Potatoes, oz........... 6 | Indian Pudding. |

*Tea.*

| | | |
|---|---|---|
| Tea, pt................. 1 | Bread, oz............... 6 | Butter, oz.............. ½ |

There are also "Beef-tea diet," "Chicken diet," "Low diet," and "Milk diet," tables, which we cannot copy for want of space.

---

SPERMATORRHŒA.—Dr. Lafont-Gouzi publishes in the *Journal de Medecine et Chirurgie* the results of a careful clinical inquiry into the effects of some medicinal agents, represented as special sedatives of sexual erethism, with a view of discovering some means of allaying genital excitement and consequent spermatorrhœa. He thinks cauterization has been too highly extolled, though doubtless applicable and efficacious in rebellious cases; but in most instances the spermatorrhœa, being the result of a too morbid energy of the organs of generation, should be treated by measures less capable of inflicting injury. He found digitaline and luguline alike inefficacious, but was more successful with the bromide of potassium, two-thirds of his cases being either cured or greatly relieved by from 15 to 30 grains being administered in two doses every afternoon for a fortnight. —*Dublin Medical Press; American Med. Times.*

